# Nervous and Mental Diseases

**Published:** 1904-03

**Authors:** 


Nervous and Mental Diseases. By Archibald Church, M. D., Professor of Nervous and Mental Diseases and Head of Neurological Department, Northwestern University Medical School, Chicago; and Frederick Peterson, M. D., President New York State Commissioner in Lunacy; Chief of Clinic, Department of Nervous Diseases, College of Physicians and Surgeons, New York. Fourth edition, thoroughly revised and enlarged. Octavo, 922 pages, with 338 illustrations: Philadelphia, New York, London: W. B. Saunders & Company. 1903. (Cloth, $5.00 net; sheep and half morocco, $6.00 net.)

The combining of the two great sciences of neurology and psychiatry in one volume has been most satisfactory to students and practitioners. Dr. Church, the author of the section on nervous diseases, has succeeded in presenting the clinical pictures of the typical nervous diseases in a clear and graphic manner. His directions for examining patients and his method of history taking are careful and minute. His summing up of the values of different signs and symptoms is modern and up-to-date. We would call particular attention to his summary on reflexes on page 37. The chapter on injuries and diseases of spinal nerves is of special value. The researches of Tizzoni, Kennedy, Bethe Ballance and Stewart in nerve regeneration are reviewed. The healing of divided nerves is a subject of great importance and one in which modern investigation has revealed the possibility of union of divided nerves after many years.

The treatment of nervous diseases is more elaborately described and more hopeful in tone than that adopted by many authors. Too often we read that "electricity may be used and hydrotherapeutic measures be tried; massage is useful." There is no such vague and meaningless phraseology to be found here. If electricity is advised, the strength and choice of current and method of application is minutely detailed, and the same is true in the directions for massage and hydrotherapy. In the disease in which the prognosis is hopeless, the physician is reminded that it is the patient and not the disease we are called upon to treat. If the disease can not be cured or its progress stayed, surely much needs to be done to make the patient more comfortable and the malady more easily borne. It is time that the hopeless helplessness of the physician in the face of disease which he cannot cure, be abandoned and that active measures for the relief of the unfortunate be substituted. Cure is not the only goal,—teaching the patient to bear his burden is fully as important.

The section of psychiatry by Dr. Peterson is a masterly condensation of the great subject of insanity. The various types of mental disease are graphically described,—the progress of the several insanities is traced in a clear and vivid manner.

The methods of examination of patients and the general treatment of insanity are excellent guides to the practitioner. The only regret we feel it a duty to record is that in this volume there is no chapter devoted to alcoholism or to drug habit. We believe these subjects should be treated in books on nervous and mental diseases, and we express the hope that a future edition will include such a chapter. We know of no book on nervous and mental diseases which is more useful to the general practitioner than the Church-Peterson. J. W. P.

